# TGFβ signaling-induced miRNA participates in autophagic regulation by targeting PRAS40 in mesenchymal subtype of glioblastoma

**DOI:** 10.20892/j.issn.2095-3941.2019.0356

**Published:** 2020-08-15

**Authors:** Yingbin Xie, Luyue Chen, Junhu Zhou, Chao Yang, Can Xu, Xiangyu Fan, Yanli Tan, Yanan Wang, Chunsheng Kang, Chuan Fang

**Affiliations:** ^1^Department of Neurosurgery, Hebei University Affiliated Hospital, Baoding 071000, China; ^2^Laboratory of Neuro-Oncology, Tianjin Neurological Institute, Department of Neurosurgery, Tianjin Medical University General Hospital and Key Laboratory of Neurotrauma, Variation, and Regeneration, Ministry of Education and Tianjin Municipal Government, Tianjin 300052, China; ^3^Department of Neurosurgery, Zhongshan Hospital Xiamen University, Xiamen 361004, China; ^4^Department of Pathology, Hebei University Affiliated Hospital, Baoding 071000, China; ^5^Affiliated Cancer Hospital and Institute of Guangzhou Medical University, Guangzhou 510095, China

**Keywords:** Autophagy, glioblastoma, microRNA, PRAS40, TGFβ signalling

## Abstract

**Objective:** Mesenchymal subtype of glioblastoma (mesGBM) is a refractory disease condition characterized by therapeutic failure and tumor recurrence. Hyperactive transforming growth factor-β (TGF-β) signaling could be a signature event in mesGBM, which leads to dysregulation of downstream targets and contribute to malignant transformation. In this study we aimed to investigate the hyperactive TGFβ signaling-mediated pathogenesis and possible downstream targets for the development of novel therapeutic interventions for mesGBM.

**Methods:** GBM-BioDP is an online resource for accessing and displaying interactive views of the TCGA GBM data set. Transcriptomic sequencing followed by bioinformatic analysis was performed to identify dysregulated microRNAs. Target prediction by MR-microT and dual luciferase reporter assay were utilized to confirm the predicted target of novel_miR56. CCK-8 assays was used to assesse cell viability. The miRNA manipulation was proceeded by cell transfection and lentivirus delivery. A plasmid expressing GFP-LC3 was introduced to visualize the formation of autophagosomes. Orthotopic GBM model was constructed for *in vivo* study.

**Results:** TGFβ1 and TGFβ receptor type II (TβRII) were exclusively upregulated in mesGBM (*P* < 0.01). Dysregulated miRNAs were identified after LY2109761 (a TβRI/II inhibitor) treatment in a mesGBM-derived cell line, and novel_miR56 was selected as a promising candidate for further functional verification. Novel_miR56 was found to potentially bind to PRAS40 via seed region complementarity in the 3′ untranslated region, and we also confirmed that PRAS40 is a direct target of novel_miR56 in glioma cells. *In vitro*, over expression of novel_miR56 in tumor cells significantly promoted proliferation and inhibited autophagy (*P* < 0.05). The expression levels of P62/SQSTM was significantly increased accompanied by the decrease of BECN1 and LC3B-II/I, which indicated that autophagic activity was reduced after novel_miR56 treatment. In addition, over expression of novel_miR56 also promoted tumor growth and inhibited autophagy *in vivo*, which is associated with worse prognosis (*P* < 0.05).

**Conclusions:** In summary, we provide novel insight into TGFβ signaling-mediated pathogenesis in mesGBM and TGFβ signaling-induced novel_miR56 may be a novel target for mesGBM management.

## Introduction

Glioblastoma (GBM) has been classified by The Cancer Genome Atlas (TCGA) research network into four major transcriptomic subtypes; of these, the mesenchymal subtype expresses signature genes functionally correlated with the process of mesenchymal transition^1^. This characteristic provides numerous potential therapeutic targets and insights into the molecular pathogenesis of GBM phenotypes. The mesenchymal transcriptomic signature can drive GBM cell aggressiveness and chemoradiotherapy resistance, leading to treatment failure and tumor recurrence, which consequently help to develop a refractory disease condition^2,3^. The overall survival of GBM patients has remained dismal for decades, and no additional successful treatment options are available to those with the refractory mesenchymal phenotype to improve the clinical outcomes^4^. Therefore, further interpretation of the mesenchymal profile of GBM and identification of the underlying mechanism responsible for malignant transition are urgently needed.

Transforming growth factor β (TGFβ) signaling is one of the most important and well-studied signaling pathways in metazoan biology. The function of this signaling pathway is highly dependent on different cell contexts. In healthy cells and precancerous cells, TGFβ signaling plays critical roles in maintaining cellular hemostasis and reversing malignant transition, while in cancer cells, this pathway sustains oncogenic transformation, such as epithelial-to-mesenchymal transition (EMT), migration, immune surveillance evasion and chemoresistance^5,6^. The TGFβ cytokine superfamily and receptor-activated mothers against decapentaplegic homologue (SMAD) cascades are the core components of TGFβ signaling, which is universally dysregulated in various types of malignancies, including GBM. For example, TGFβ1, an upregulated mesenchymal signature gene, mediates mesenchymal transition and poor prognosis in GBM^2,3^. However, phase II clinical trials of galunisertib^7^, a TGFβ receptor type I (TβRI) inhibitor, and trabedersen^8^, a TGFβ2 inhibitor, failed to improve the overall survival of GBM patients, which suggests the need for further understanding of the pathological implications of TGFβ signaling in GBM. The SMAD transcription factor-mediated gene profile has been unveiled after years of intense exploration, but most of the efforts focused on protein-coding effectors. With the increasing emphasis on the biological implication of non-coding RNAs (ncRNAs), it is essential to profile novel non-protein-coding effectors to supplement our knowledge of TGFβ signaling-mediated pathogenesis. The discovery of TGFβ signaling-regulated microRNAs (miRNAs), the best-understood class of ncRNAs, has supported this concept^9^. MiRNAs are 15-27 nt-long single-stranded ncRNAs that mediate mRNA degradation or inhibit translation initiation by seed (guide positions 2-8)-mediated complementation of the 3′ untranslated region (3′-UTR)^10^. The expression of miR-182 is induced by TGFβ-SMAD binding to the *MIR182* promoter, and miR-182 upregulation thus sustains NFκB activation in glioma subsets^11^. In addition, SMAD proteins control DROSHA-mediated miR-21 maturation at the post-transcriptional level^12^. It is thus suggested that dysregulated TGFβ signaling may cause the deregulation of a subset of miRNAs as well, and some of those previously unnoticed functional miRNAs within the subset could be responsible for the genesis of certain malignant phenotypes.

Autophagy (which here refers to the best-studied type, macroautophagy) is an evolutionarily conserved process by which eukaryotic cells maintain intracellular homeostasis and adapt themselves to cellular stress to survive. Autophagy activities, such as sequestration of endogenous or exogenous cytoplasmic waste and subsequent lysosomal degradation, are tightly controlled by a set of signaling events^13^. Mechanistic target of rapamycin complex 1 (mTORC1) and the BECN1-phosphatidylinositol 3 kinase catalytic subunit type 3 (PIK3C3) complex are two main signaling checkpoints that turn autophagy off and on, respectively, and dysregulated pathways influencing these two checkpoints have been linked with various disease conditions, including GBM^14^. Emerging studies agree that deregulated autophagic activities may result in dichotomous consequences of self-consumption or self-adaption^13,15,16^. However, autophagy-related determination of cell fate is still a factor. Autophagic death is the major GBM cell death pathway because of its ability to resist apoptotic stimuli, but the underlying mechanism remains unclear^17^. Knowledge of the role of autophagy in GBM pathogenesis has just begun to solidify, and a better understanding of protective and lethal autophagic activities will contribute to mapping the context-dependent functional network, which underlies the molecular basis for selectively targeting autophagy in GBM.

In the present study, we noted that in TCGA GBM data, TGFβ1 and TβRII, two of the 840 signature genes for GBM subtyping^1^, were exclusively upregulated in mesenchymal subtype of GBM (mesGBM). In a cell line derived from a patient-derived tumor xenograft (PDX) of mesenchymal GBM (PDXM), a set of TGFβ signaling-associated miRNAs was identified after LY2109761 (a TβRI/II inhibitor)^18^ treatment by transcriptomic sequencing. Interestingly, some of these miRNAs were previously unreported miRNAs, which cannot be found in the miRbase database and were temporarily named “novel_miR”. Among those differentially expressed miRNAs, novel_miR56 was chosen as a candidate for functional and mechanistic validation for TGFβ signaling-mediated pathogenesis. Proline-rich akt substrate of 40 kDa (PRAS40), a suppressor of mTORC1 activity^19,20^, was experimentally validated as a direct target of novel_miR56, which incorporated TGFβ signaling-induced miRNA into autophagic regulation signaling. Novel_miR56 upregulation impaired autophagic activity and promoted the survival capability of GBM cells. Collectively, we demonstrate a previously unreported mechanism of TGFβ signaling-mediated autophagic regulation, which possibly contributes to the formation of GBM mesenchymal subtypes.

## Materials and methods

### Cell culture

Human GBM (U87, LN229 and U251), breast cancer (M231) and renal cancer (ACHN) cell lines were purchased from the China Academia Sinica Cell Repository. Two newly established GBM cell lines, U87-EGFRvIII, a U87 cell line stably expressing EGFRvIII, and N9/N33, were kind gifts from Prof. Huan Ren (Harbin Medical University) and Prof. Tao Jiang (Beijing Tiantan Hospital), respectively. All cell lines were maintained in appropriate culture media (DMEM or RPMI-1640) supplemented with 10% fetal bovine serum (Gibco), as well as with 0.4 g/mL G418 (EMD Bioscience) for U87-EGFRvIII cells, at 37 °C in a 5% CO_2_ humidified chamber.

### Quantitative real-time PCR (qRT-PCR)

Total RNA was extracted using TRIzol (Invitrogen). qRT-PCR assays were performed to measure the expression levels of novel_miR26/56/93/97/119 and PRAS40 according to the manufacturer’s instructions for SYBR Green PCR Master Mix (Promega). Real-time quantification to measure novel miRNAs was performed with a Hairpin-it™ miRNAs qPCR Quantitation Kit (GenePharma). GAPDH and RNU6 were employed for the normalization and quantification of PRAS40 and miRNA, respectively. Real-time PCR was performed using the DNA Engine Opticon 2 Two-Color Real-Time PCR detection system (Bio-Rad Laboratories) in the presence of SYBR Green. Real-time PCR data were analyzed by the comparative Ct method^21^. The following gene-specific primers were used: novel_miR26 (F: 5′-CGCCGTTGCAGCTGC-3′; R: 5′-CAGAGCAGGGTCCGAGGTA-3′); novel_miR56 (F: 5′-CCGTTGGTAGTGTGGCCG-3′; R: 5′-TATGGTTGTTC TGCTCTCTGTCTC-3′); novel_miR93 (F: 5′-ATATCC GGCCACGCGC-3′; R: 5′-TATGGTTGTTCACGACTCC TTCAC-3′); novel_miR97 (F: 5′-GGTCCCATCGGGTT GCTT-3′; R: 5′-TATGGTTGTTCACGACTCCTTCAC-3′); novel_miR119 (F: 5′-GTTCCCATGTAGTCGTGGCC-3′; R: 5′-TATGGTTGTTCACGACTCCTTCAC-3′); PRAS40 (F: 5′-ATGGCTACCGCGTTCATGCT-3′; R: 5′-ATCCGCGCCC CTTCAGCTT-3′); GAPDH (F: 5′-CTCAAGGGCAT CCTGGGCTAC-3′; R: 5′-CAGCCCCAGCGTCAAAGGT-3′); and RNU6 (F: 5′-ATTGGAACGATAC-AGAGAAGATT-3′; R: 5′-GGAACGCTTCACGAATTTG-3′).

### Cell transfection

Transfection was performed using Opti-MEM medium and Lipofectamine 3000 (Invitrogen) according to the manufacturer’s protocol. Novel_miR56 mimics (5′-GGUAGUGUGGCCGAGCGGUCU-3′) and inhibitors (5′-AGACCGCUCGGCCACACUACC-3′) were synthesized and purified by Invitrogen (Beijing, China). A PRAS40 3′-UTR reporter plasmid was constructed by inserting the two predicted novel_miR56 binding sites (5′-GGGCCGCGUCCGCC CCGUCCCACACUACG-3′ and 5′-GGCCUUCAAUUU ACGUUCUUUACACUACG-3′) into the 3′-UTR of a promoter-driven luciferase gene. The GFP-LC3 plasmid was used to measure the number of autophagosomes, which is indicated by the formation of GFP-positive granules.

### Lentivirus preparation and *in vitro* infection

Lentiviral vectors expressing nonsense control, novel_miR56 (5′-GGUAGUGUGGCCGAGCGGUCU-3′) or luciferase were all generated by GenePharma. Cell infections were carried out according to GenePharma’s recommendations.

### Western blot analyses and immunostaining

Western blot, immunohistochemistry (IHC) and immunofluorescence (IF) assays were performed as previously described^22^. Antibodies for PRAS40, BECN1, LC3B, Ki-67, SQSTM1, mTOR, LAMP1, GAPDH and β-tubulin were all purchased from Cell Signaling Technology (CST), and the expression of GAPDH and β-tubulin were used as internal controls.

### Cell viability

Cell viability was assessed using the Cell-Counting Kit 8 (CCK8, Dojindo Laboratories) assay according to the manufacturer’s instructions. In brief, cells were seeded at 5,000 cells per well in 96-well plates and incubated for the appropriate length of time in an incubator after treatment. CCK8 solution was added to each well of the plate and incubated for 4 h, and a microplate reader was used to measure the absorbance at 450 nm.

### PDX and orthotopic GBM models

Creation of the mesGBM PDX mouse model started with the harvest of pathologically confirmed human glioblastoma specimens from the operating room. The pathological tissue specimens and animals involved in the study were subject to approval by the Affiliated Hospital of Hebei University and the Institute of Animal Care and Use Committee at Tianjin Medical University (Approval No. HDFY-KL-LL-2018-17, TMUaMEC 2018034). Subsequently, RNA extraction was performed, and transcriptomic sequencing was used to classify the clinical GBM samples into four transcriptomic subtypes (neural, proneural, classical, and mesenchymal) based on the 840-gene transcriptomic signature^1^. The mesGBM specimens were then implanted subcutaneously into the flanks of female SCID mice, while a portion of each implanted specimen was cryopreserved for long-term storage. The xenografts were passaged or collected when the tumor volume exceeded 1 cm^3,23^. For the establishment of the orthotopic mesGBM model, the harvested xenografts were digested into single-cell suspensions. The orthotopic tumor models were constructed as previously described^24^. Paraffin-embedded sections (8 mm) were stained with hematoxylin and eosin (H&E). The animal studies were performed according to internationally recognized guidelines for animal welfare and experimental regulations. Survival curves were plotted according to the Kaplan-Meier method.

### Statistical analysis

The data are presented as the means ± S.E.M. of three independent experiments or the means ± S.D. of samples assayed in triplicate. Statistical comparisons were made by Student’s *t*-test. A *P*-value < 0.05 was considered statistically significant.

## Results

### TGFβ signaling is activated in mesGBM

**Figure 1:**
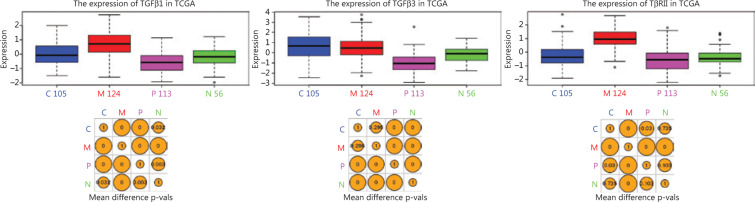
Visualization of the expression levels of TGFβ1/3 and TβRII, 3 of the 840 transcriptomic signature genes for GBM subtyping^1^, was achieved using the Glioblastoma Bio Discovery Portal (GBM-BioDP) (https://gbm-biodp.nci.nih.gov/).

TGFβ signaling plays an important role in mesenchymal transition in cancer, and TGFβ1/3 and TβRII are the three TGFβ signaling genes within the list of 840 transcriptomic signature genes that are used to classify GBM subtypes. Using the Glioblastoma Bio Discovery Portal (GBM-BioDP), an online resource for accessing and displaying interactive views of the TCGA GBM data set, we visualized the expression levels of TGFβ1/3 and TβRII in the four GBM subtypes. TGFβ1 and TβRII were exclusively upregulated in the mesenchymal subtype (*P* < 0.01), while TGFβ3 was downregulated in proneural and neural subtype (**Figure 1**). These results suggested that hyperactive TGFβ signaling could be a signature event in mesGBM.

### Novel_miR56 is induced by dysregulated TGFβ signaling in mesGBM

**Figure 2:**
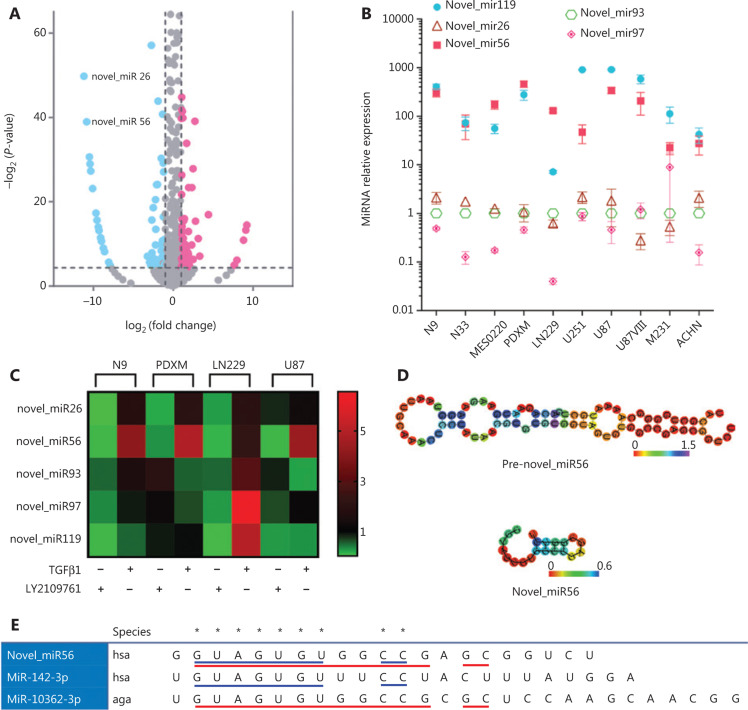
TGFβ pathway-associated miRNAs include some previously unreported miRNAs. (A) Volcano plot illustrating differentially expressed miRNAs after LY2109761 treatment in PDXM cells with a fold-change cut-off of > 2 or < 0.5 and a significant *P*-value of < 0.05. The data were obtained from transcriptomic sequencing. (B) The expression levels of novel_miR26/56/93/97/119 were detected in selected cancer cell lines and a mesGBM specimen (MES0220). The error bars are presented as the means ± s.e.m. from three independent experiments. (C) The changes in novel_miR26/56/93/97/119 expression were detected by qRT-PCR in GBM cell lines (N9, PDXM, LN229 and U87) after manipulation of TGFβ pathway activity with LY2109761 or TGFβ1; the changes are visualized by a double gradient heat map. (D) The secondary structures of pre-novel_miR56 and mature novel_miR56 were predicted with RNAfold. (E) Novel_miR56 shared an identical seed region with hsa-miR-142-3p (blue underlining) and aga-miR-10362-3p (red underlining). qRT-PCR data are normalized to the fold-change in RNU6.

TGFβ signaling dysregulation may lead to dysregulated downstream miRNAs. To identify TGFβ signaling-regulated miRNAs in mesGBM, PDXM cells were treated with DMSO or LY2109761 before transcriptomic sequencing. The miRDeep*^25^ strategy was applied to predict novel miRNAs, and 113 differentially expressed miRNAs were identified with a fold-change cut-off of > 2 or < 0.5 and a significant *P*-value of < 0.05; of these, 28 potentially novel miRNAs could not be found in the miRBase database. There were 60 potential direct target miRNAs of TGFβ signaling that were downregulated after LY2109761 treatment (**Figure 2A**). Deactivation of TGFβ signaling resulted in the downregulation of its downstream miRNA effectors, and interestingly, the top 20 most significantly downregulated miRNAs contained 17 unreported miRNAs, indicating previously ignored functional implications of these potential miRNAs and the significant value of their functional evaluation (data not shown). In addition, the expression of five potential novel miRNAs, novel_miR26/56/93/97/119, was detected in all tested cell lines (N9, N33, MES0220, PDXM, LN229, U251, U87, U87VIII, M231 and ACHN), indicating that these novel miRNAs were not a cell type-specific phenomenon (**Figure 2B**). In particular, a relatively higher abundance of novel_miR56/119 was found in these cell lines. Recombinant TGFβ1 protein and LY2109761 treatment, which modulated TGFβ signaling activity, could alter the expression of novel_miR26/56/93/97/119 in the N9, PDXM, LN229 and U87 cell lines (**Figure 2C**). However, the changes in novel miRNA expression in response to these modulators were not consistent among different cell lines and miRNAs, and only novel_miR56 was consistently and positively correlated with TGFβ signaling activity. Therefore, novel_miR56 may be the most promising candidate for further functional validation of TGFβ signaling-induced novel miRNAs.

Prediction of the secondary structure of the novel_miR56 precursor (pre-novel_miR56) by RNAfold demonstrated that pre-novel_miR56 harbored the typical stem-loop structure of a miRNA precursor (**Figure 2D**). Further comparison of the RNA sequence of mature novel_miR56 with miRBase-documented miRNAs revealed that novel_miR56 shared the same seed region (guide position 2-8) with hsa-miR-142-3p and aga-miR-10362-3p, indicating that novel_miR56 might belong to the hsa-miR-142 family. An identical sequence at guide positions 2-13 and 15-16 in novel_miR56 and aga-miR-10362-3p supported this structural feasibility (**Figure 2E**). Therefore, novel_miR56 could be a previously unreported miRNA that serves as a functional effector of TGFβ signaling in mesGBM.

### PRAS40 is an autophagy-associated functional target of novel_miR56

**Figure 3:**
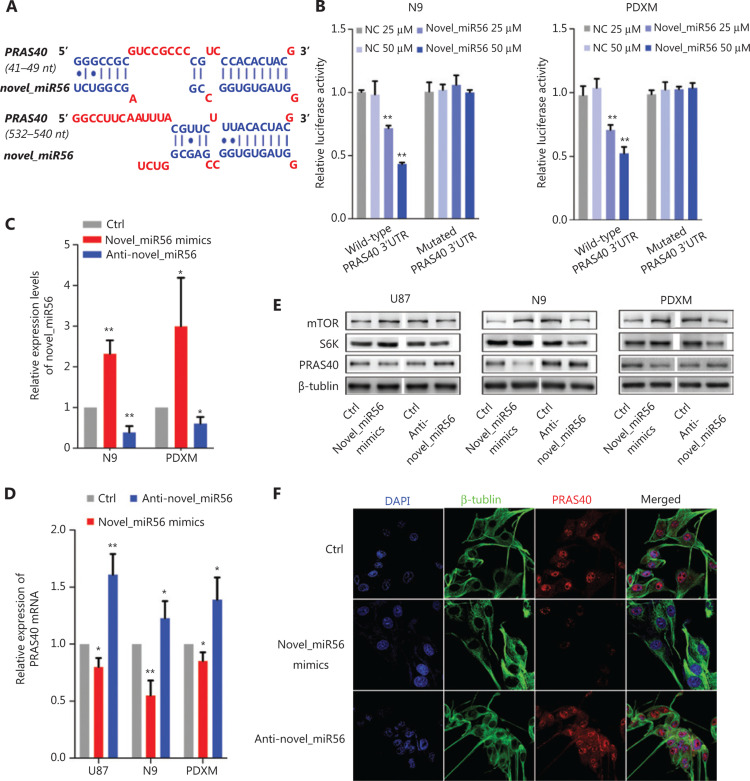
Novel_miR56 is a functional miRNA that targets PRAS40. (A) Two binding sites of novel_miR56 on the PRAS40 3′-UTR were predicted with an online prediction method, MR-microT. (B) A reporter gene assay was performed using PDXM and N9 to confirm the interaction of novel_miR56 and its predicted binding sites on the PRAS40 3′-UTR using plasmid constructs containing wild-type or mutated PRAS40 3′-UTR binding sites in the 3′-UTR of the luciferase gene. (C) The efficacy of novel_miR56 mimics and inhibitor (anti-novel_miR56) to manipulate novel_miR56 were experimentally confirmed. The expression levels of PRAS40 mRNA (D) and protein (E) were negatively correlated with the manipulation of novel_miR56 expression in U87, N9 and PDXM cells. S6K and mTOR expression was inversely correlated with PRAS40 after novel_miR56 intervention. (F) Immunofluorescence and western blotting showed similar trends for PRAS40 expression changes in response to novel_miR56 manipulation in PDXM cells. The error bars are presented as the means ± S.E.M. from three independent experiments. qRT-PCR data are normalized to the fold-change in RNU6. *P < 0.05, **P < 0.01.

Direct targets of novel_miR56 were predicted based on sequence by an online prediction method, MR-microT. In searching for potential novel_miR56 targets involved in autophagic regulation, we focused on the gene subset correlated with autophagic activity, and PRAS40, an inhibitor of mTORC1 activity, was chosen as a potential target due to its two predicted binding sites at 41-49 bp and 532-540 bp of the 3′-UTR (**Figure 3A**). To exogenously modulate the expression of novel_miR56, we employed artificial novel_miR56 mimics and inhibitors, which were experimentally validated to increase or decrease novel_miR56 in the N9 and PDXM cell lines (**Figure 3C**, *P* < 0.05). The novel_miR56 mimics reduced the luciferase activity of the wild-type PRAS40 3′-UTR-expressing reporter in a concentration-dependent manner in the N9 and PDXM cell lines, while no significant change in the luciferase activity was observed in cells transfected with the reporter containing the mutated novel-miR56 binding sites; these results demonstrated the binding between the PRAS40 3′UTR and novel_miR56 (**Figure 3B**, *P* < 0.01). The alterations in PRAS40 mRNA and protein levels in response to novel_miR56 intervention further supported that PRAS40 is a direct target of novel_miR56 (**Figure 3D,E**). S6K (S6 kinase) and mTOR expression was inversely correlated with PRAS40 after novel_miR56 intervention (**Figure 3E**). Additionally, the change in the immunofluorescent signal intensity of PRAS40 in PDXM cells after novel_miR56 intervention shared a similar trend with its counterpart according to western blot results (**Figure 3F**). Collectively, we proved that novel_miR56 can bind to the PRAS40 3′-UTR as predicted and reduce the protein level, which suggests that PRAS40 may act as a functional target for TGFβ signaling-mediated autophagic regulation.

### Novel_miR56 inhibits autophagic activity but promotes survival in GBM *in vitro*

**Figure 4:**
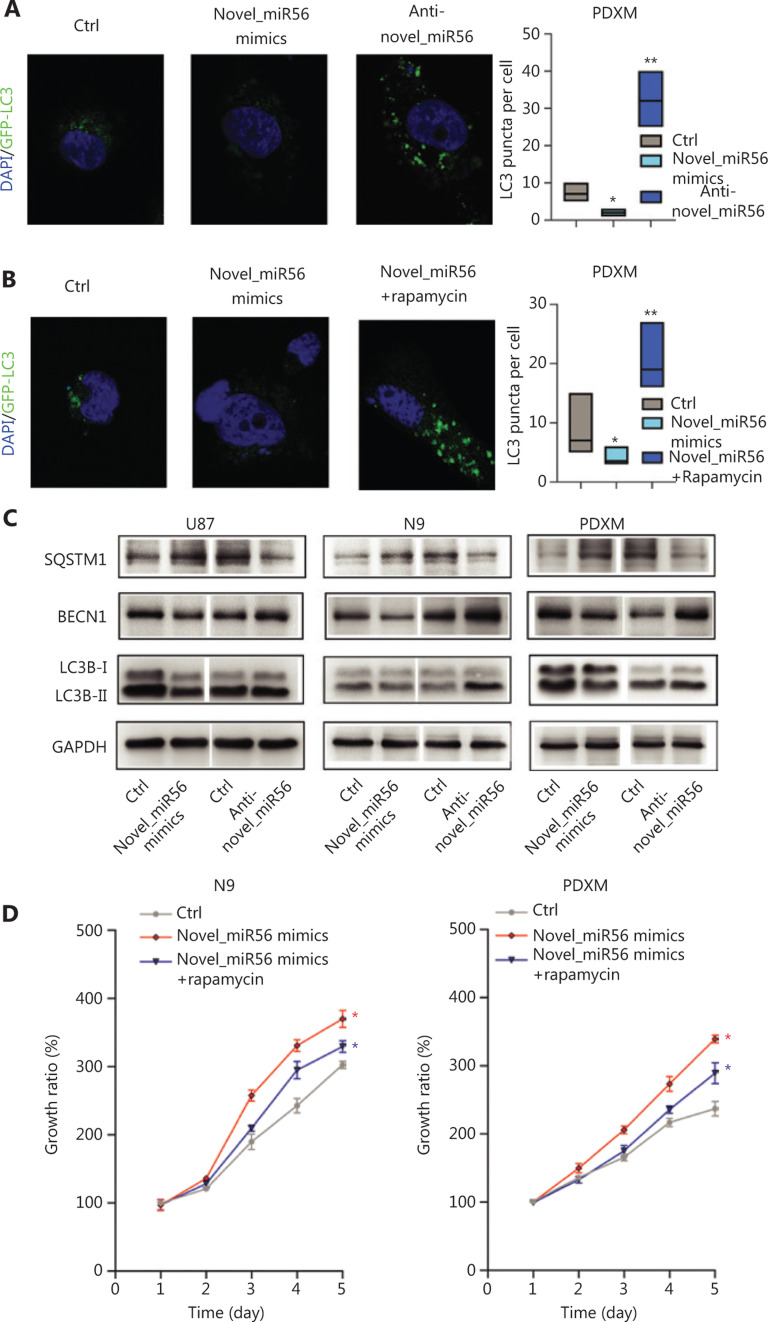
Novel_miR56 is implicated in autophagic regulation by targeting PRAS40. (A) Altered novel_miR56 expression affected the autophagic activity in PDXM cells stably expressing GFP-LC3, (B) and rapamycin restored the autophagic activity of novel_miR56-mediated inhibition of autophagy. Autophagic activity was indicated by the accumulation of GFP-enriched subcellular granules around the nucleus and LC3 puncta per cell was calculated via Image J. (C) Western blot was performed to detect the expression levels of the autophagy-associated proteins P62/SQSTM1, BECN1 and LC3B-I/II. GAPDH was used as an internal control. (D) CCK8 assays were applied to evaluate cell growth in response to novel_miR56 or novel_miR56 plus rapamycin treatment in N9 and PDXM cells. The error bars are presented as the means ± S.E.M. from three independent experiments. *P < 0.05.

PRAS40 has been identified as a partner and inhibitor of mTORC1 as it is an important checkpoint in stopping autophagic initiation. In PDXM cells stably expressing GFP-LC3, an intracellular green fluorescent label recombinant protein for autophagosomes, exogenous upregulation of novel_miR56, a miRNA regulator of PRAS40, resulted in a significant decrease in the number of GFP-LC3-enriched subcellular granules around the nucleus, which indicated decreased autophagic activity in PDXM cells in response to increased novel_miR56 expression. In contrast, reduced expression of novel_miR56 increased the number of GFP-LC3-labeled granules (**Figure 4A**). Rapamycin, a functional partner of FK506-binding protein 12 (FKBP12) that binds to and inhibits the mTOR protein of mTORC1, restored autophagic activity, supporting the assumption that activated mTORC1 mediates the novel_miR56-induced inhibition of autophagy (**Figure 4B**)^26^. LC3B, a member of the mammalian homologs of yeast Atg8, mostly involved in autophagosome formation and the conversion of LC3-I (cytosolic form) into LC3-II (membrane-bound lipid form) is the characteristic signature of autophagic membranes^13^. The increase in expression of P62/SQSTM1 and decrease in BECN1 and LC3B-II/I were in accordance with reduced autophagic activity after novel_miR56 treatment (**Figure 4C**). Although novel_miR56-mediated inhibition of autophagy was observed in GBM cells *in vitro*, the biological influence on GBM cells was unclear due to the dichotomous effect of autophagy. Autophagy plays an important role in GBM cell death, and cell survival ability was measured by CCK8 assay. Surprisingly, novel_miR56 overexpression promoted GBM cell growth, and this pro-survival effect could be partially abolished by rapamycin. These data suggested that novel_miR56-mediated autophagic inhibition could sustain the proliferative phenotype of GBM and that mTORC1 is an important mediator responsible for this oncogenic process (**Figure 4D**, *P* < 0.05). These results demonstrated that novel_miR56 could reactivate mTORC1-mediated autophagy inhibition by targeting PRAS40, and the reduced autophagic activity led to a proliferative phenotype in GBM.

### Novel_miR56 overexpression boosts mesGBM cell growth *in vivo*

**Figure 5:**
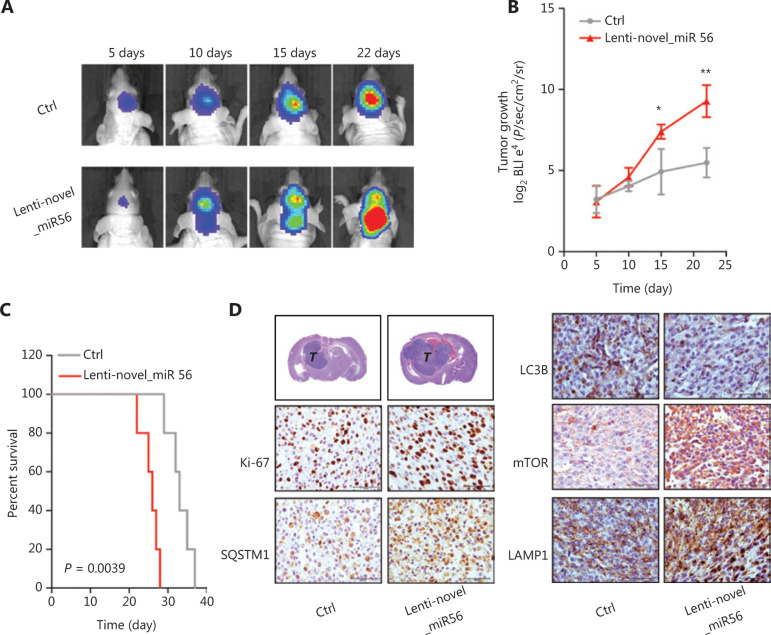
Novel_miR56 promotes tumorigenesis *in vivo.* (A) Representative *in vivo* images of mice were obtained on days 5, 10, 15, and 22 after implantation. Novel_miR56 overexpression was achieved by introducing lentiviruses expressing the mature novel_miR56 sequence (lenti-novel_miR56). (B) Quantitative analysis of *in vivo* tumor growth revealed that overexpression of novel_miR56 promotes tumor growth (*n* = 5). (C) Animal survival analysis [*n* = 10, log-rank (Mantel-Cox) test]. (D) Histological staining of the orthotopic tumor samples. The scale bar corresponds to 50 μm. The error bars are presented as the means ± s.e.m. from three independent experiments. *P < 0.05, **P < 0.01.

Our data showed that novel_miR56 overexpression promotes GBM cell survival *in vitro*, but whether novel_miR56 functioned similarly *in vivo* was unknown. An orthotopic mesGBM PDX mouse model was applied to evaluate the biological function of novel_miR56 *in vivo*. Prior to implantation, PDXM cells were infected with lentiviruses expressing luciferase and novel_miR56 or scrambled control for 48 h, and these pre-treated cells were then injected orthotopically into nude mice. *In vivo* imaging of the mice at days 5, 10, 15, and 22, followed by luminescent quantification, indicated that forced expression of novel_miR56 increased the growth of mesGBM orthotopic tumors (**Figure 5A,B**). The survival times were shorter in mice bearing novel_miR56-expressing orthotopic tumors than in those bearing control sequence-expressing orthotopic tumors (**Figure 5C**, *P* = 0.0039). Histopathological staining of mesGBM orthotopic tumors revealed larger tumor volumes and a higher ki67 index in novel_miR56-treated mice. Besides, a hyper-staining of SQSTM1, mTOR, and LAMP1, and a hypo-staining of LC3B were observed in orthotopic GBM specimens overexpressing novel_miR56 (**Figure 5D**). The accumulation of intracellular SQSTM1, a selective autophagy substrate and cargo receptor for degradation of ubiquitinated substrates, and LAMP1, a major lysosomal membrane protein, could be the result of impaired autophagosome formation and lysosome consumption. Altogether, these findings revealed that novel_miR56 enhances mesGBM cell growth *in vivo* and that the underlying mechanism may be attributed to PRAS40 suppression and mTORC1 reactivation, which agrees with *in vitro* findings on the pro-survival role of novel_miR56 in GBM.

## Discussion

GBM is the most common and lethal primary malignant brain tumor. Despite advances in molecular pathogenesis in recent years that have advanced our understanding of the biology of this disease, little progress has been made to change the poor prognosis, even in the era of molecular medicine^4^. MesGBM is subclassified by a subset of transcriptomic signature genes associated with mesenchymal transition and characterized by aggressiveness and resistance to chemoradiotherapies. The mesenchymal signature was considered to contain promising targets for mesGBM; however, after nearly a decade, few successful drugs have been developed for this disease, which thus far remains fatal. To improve the overall survival of GBM patients, or at least patients with the refractory mesenchymal subtype, new insights could be provided by further deepening our knowledge of malignant transformation.

In our study, to search for novel insight into mesGBM pathogenesis, we reviewed the transcriptomic signature genes of mesGBM and found the exclusive upregulation of TGFβ1 and TβRII in the mesenchymal subtype and downregulation of TGFβ3 in the proneural subtype in the TCGA GBM dataset. TGFβ1/3 and TβRII are three important constituents that activate TGFβ signaling. Thus, hyperactive TGFβ signaling could be a signature event in mesGBM. TGFβ signaling is a multifunctional pathway that regulates a variety of oncogenic processes in cancer cells, and it has been reported to correlate with mesenchymal transition and poor prognosis in GBM^2,3^. However, trials for drugs targeting TGFβ signaling fail to achieve clinical benefit in patients with GBM, which therefore urges us to reevaluate the pathological relevance of TGFβ signaling in mesGBM. Although the importance of understanding TGFβ signaling has been attached to cancers such as GBM for years, most research efforts were dedicated to protein-coding genes. There is an emerging consensus that ncRNAs are key players in epigenetic regulation, and miRNAs are the most well-studied class of ncRNAs that play functional roles in post-transcriptional regulation. Mature miRNAs are encoded by the genome and undergo step-by-step maturation, which is strictly coordinated by multiple pathways, including TGFβ signaling pathways. TGFβ signaling crosstalks with miRNA machinery at the level of miRNA transcription and DROSHA processing, together increasing expression levels. miRNAs are promising emerging targets for drug development in brain tumors^27^. The exclusively hyperactive TGFβ signaling in mesGBM may lead to downstream miRNA dysregulation, which will provide numerous potential therapeutic targets for treating mesGBM. We used LY2109761, a TβRI/II inhibitor, to inhibit TGFβ signaling activity in a PDXM cell line derived from a mesGBM PDX model and performed transcriptome sequencing on LY2109761-treated and control cells. There were 113 miRNAs with differential expression in cells after LY2109761 treatment, including 28 unreported miRNAs. These unreported miRNAs, temporarily named novel_miRs, were considered unregistered miRNAs in miRBase. The miRNAome expands gradually over time due to advancements in technology and algorithms of miRNA mining, and the latest version of miRBase (version 22) contains 1917 miRNA precursors and 2654 mature miRNAs for homo sapiens, with an additional 36 miRNA precursors and 66 mature miRNAs compared to the previous version (version 21). Therefore, it is inevitable that novel miRNAs will be discovered, and these miRNAs may play previously unknown roles in particular disease processes, such as the TGFβ signaling-mediated malignant transition of mesGBM, as shown in this study. Here, we paid special attention to the downregulated novel miRNAs that may be direct downstream effectors of TGFβ signaling, and we sought novel insight into mesGBM pathogenesis. Five novel miRNAs (novel_miR26/56/93/97/119) were selected for validation in GBM, mesGBM, breast cancer and renal cancer cell lines. Among these novel miRNAs, novel_miR56 was chosen for functional evaluation due to its relatively higher abundance and consistently positive correlation with TGFβ signaling activity in GBM cell lines.

To better understand the functional implications of novel_miR56 in TGFβ signaling-mediated pathogenesis, the functional targets of novel_miR56 should be verified. However, most of the online miRNA target prediction tools are only compatible with miRbase-registered miRNAs, and there are limited options for sequence-based predictions. Here, we used MR-microT, an open access online tool provided by the DIANA lab, to predict novel_miR56 targets. In this study, due to our special interest in autophagy in GBM, PRAS40 was chosen and experimentally validated as a functional target that participates in autophagic regulation by suppressing mTORC1 activity^19,20^. Autophagy in cancer is a rapidly growing research field, and recent studies suggest the possibility of targeting autophagy to fight cancers^28^. In GBM, due to insensitivity to apoptotic stimuli, autophagy is the major pathway that controls cell death^17^, and it is possible that dysregulated autophagy can sustain tumor cell survival. TGFβ signaling has been implicated in autophagic regulation in glioma^29,30^, but the downstream effectors of TGFβ signaling that crosstalk with the autophagic machinery remain unclear. mTORC1 is an established inhibitor of autophagy induction^13,31^, and PRAS40 inhibition by TGFβ signaling-induced novel_miR56 reactivates mTORC1, resulting in autophagy suppression. Our results first link TGFβ signaling-induced miRNA with autophagic regulation in mesGBM.

Activation or deactivation of autophagy has opposing, context-dependent roles in cancers, including GBM. In this study, novel_miR56-mediated autophagic inhibition sustained cell growth in GBM cells as well as mesGBM cells, indicating that TGFβ signaling can induce the expression of certain miRNAs to suppress autophagic activity. One of the major reasons for targeting autophagy in cancer is the cytoprotective role in response to various conditions of cellular stress^13,28^; however, emerging evidence in recent years suggests that autophagy can mediate tumor suppression via inducing cellular senescence and reducing chromosomal instability^32^. Rampant genomic instability is one of the hallmark features of GBM, and autophagy suppression mediated by TGFβ signaling-induced novel_miR56 expression may abolish the self-mediating capability to restore chromosomal stability. This process may provide novel insight into mechanism of proliferation maintenance in mesGBM by linking autophagic activity with chromosomal stability regulation.

## Conclusions

**Figure 6:**
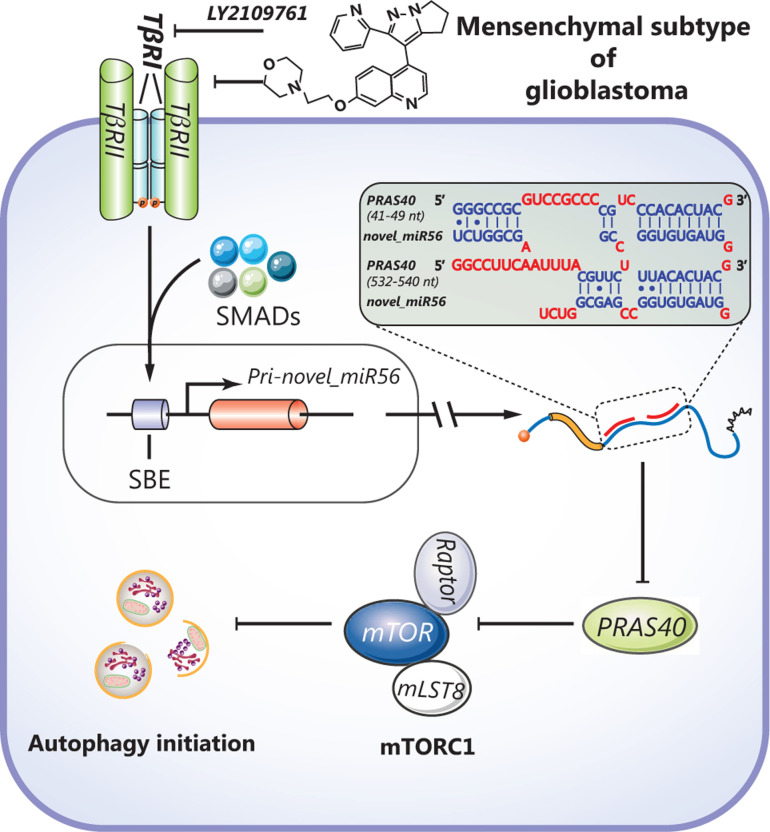
A schematic diagram illustrates the regulation of autophagy by the TGFβ pathway-induced novel miR56 in mesGBM. Novel miR56, a downstream target of TGFβ pathway, directly targets PRAS40 by miRNA-mRNA interaction at the post-transcriptional level. The activity of mTORC1, an important check point of autophagic initiation, is suppressed by PRAS40. Induction of novel_miR56, inhibition of PRAS40 and deactivation of mTORC1 are the underlying mechanism for the TGFβ pathway-mediated pathogenesis in mesGBM.

In summary, our study reveals a novel mechanism for TGFβ signaling-mediated autophagic regulation in GBM. TGFβ signaling-induced novel_miR56 can directly target PRAS40 and reactivate mTORC1 activity, resulting in autophagy suppression but survival maintenance (**Figure 6**). The oncogenic role of TGFβ signaling-induced novel_miR56 provides novel insight into mesGBM formation and a molecular basis for drug development.
